# Astragaloside IV improves lipid metabolism in obese mice by alleviation of leptin resistance and regulation of thermogenic network

**DOI:** 10.1038/srep30190

**Published:** 2016-07-22

**Authors:** Hui Wu, Yan Gao, Hai-Lian Shi, Li-Yue Qin, Fei Huang, Yun-Yi Lan, Bei-Bei Zhang, Zhi-Bi Hu, Xiao-Jun Wu

**Affiliations:** 1 Shanghai Key Laboratory of Complex Prescription, The Ministry of Education (MOE) Key Laboratory for Standardization of Chinese Medicines, Institute of Chinese Materia Medica, Shanghai University of Traditional Chinese Medicine, Shanghai, 201203, China

## Abstract

Obesity is a worldwide threat to public health in modern society, which may result from leptin resistance and disorder of thermogenesis. The present study investigated whether astragaloside IV (ASI) could prevent obesity in high-fat diet (HFD)-fed and db/db mice. In HFD-fed mice, ASI prevented body weight gain, lowered serum triglyceride and total cholesterol levels, mitigated liver lipid accumulation, reduced fat tissues and decreased the enlargement of adipose cells. In metabolic chambers, ASI lessened appetite of the mice, decreased their respiratory exchange ratio and elevated VCO_2_ and VO_2_ without altering circadian motor activity. Moreover, ASI modulated thermogenesis associated gene expressions in liver and brawn fat tissues, as well as leptin resistance evidenced by altered expressions of leptin, leptin receptor (ObR) or appetite associated genes. In SH-SY5Y cells, ASI enhanced leptin signaling transduction. However, in db/db mice, ASI did not change body weight gain and appetite associated genes. But it decreased serum triglyceride and total cholesterol levels as well as liver triglyceride. Meanwhile, it significantly modulated gene expressions of PPARα, PGC1-α, UCP2, ACC, SCD1, LPL, AP2, CD36 and SREBP-1c. Collectively, our study suggested that ASI could efficiently improve lipid metabolism in obese mice probably through enhancing leptin sensitivity and modulating thermogenic network.

Obesity has become recognized as a worldwide health threat and a major public health challenge. It is mainly characterized by an excessive increase in adipose tissue mass and dysregulation of lipid metabolism^1^ and is a serious risk factor for many disorders such as hypertension^2^, cardiovascular disease, type-2 diabetes^3^ and chronic metabolic disease^4^.

Leptin resistance has been known to be closely associated with obesity^5^. Leptin is a key hormone produced primarily in adipose tissue and involved in the regulation of food intake and energy expenditure^6^. It signals through leptin receptor b (ObRb), one of the splicing isoforms, to exert the main physiological action^7^. Binding of leptin to ObRb activates Janus tyrosine kinase 2, leading to the phosphorylation of signal transducer and activator of transcription 3 (STAT3), and thus triggers downstream cascades^8^. While ObRa, another isoform of leptin receptor, is closely associated with the transportation of circulating leptin at blood-brain barrier^9^. Although the mechanisms of leptin resistance remain unclear, aberrant leptin uptake, disrupted leptin signaling cascades, and decreased leptin receptors in central nervous system are proposed to be the possible reasons^10^,^11^. Therefore, drugs facilitating the alleviation of leptin resistance may benefit the treatment of obesity.

*Astragali Radix*, one of the most commonly used traditional Chinese medicine (TCM), is prepared from the roots of *Astragalus membranaceus* (Fisch) Bunge or *Astragalus mongholicus* Bunge. It is widely used in coffee, tea substitutes and food, in places such as Europe, the Middle East and Asia^12^,^13^. Currently, the products of *Astragali Radix*, such as Astragali tea and over-the-counter dietary supplement capsules, are available in U.S. health food markets^14^. In China, Astragalus-jujube-wolfberry tea is a well-known anti-cancer tea that can enhance body vitality and immunity^15^. Moreover, *A. Radix* has many therapeutic functions, including immunoregulatory, antioxidant, anti-cancer, antiviral, diuretic, hypolipidemic, and hypoglycemic effects^16^,^17^. Astragaloside IV (ASI), one of the major and active components of *A. Radix*, has been shown to possess many pharmacological activities including anti-oxidation^18^, anti-hypertension^19^, anti-diabetes^20^, and anti-inflammation^21^,^22^. However, little is known about this compound in the improvement of leptin resistance and associated thermogenetic network. Here, the effects of ASI on prevention of the development of hyperlipidemia and alleviation of leptin resistance in high-fat diet (HFD)-fed C57BL/6 mice and db/db mice were investigated and the possible underlying mechanisms were discussed. Our results implicated the potential application of ASI and its derivatives in the prevention of obesity as anti-leptin resistance agents.

## Results

### ASI prevented body weight gain and reduced accumulation of fat tissues in HFD fed mice

**Figure 1:**
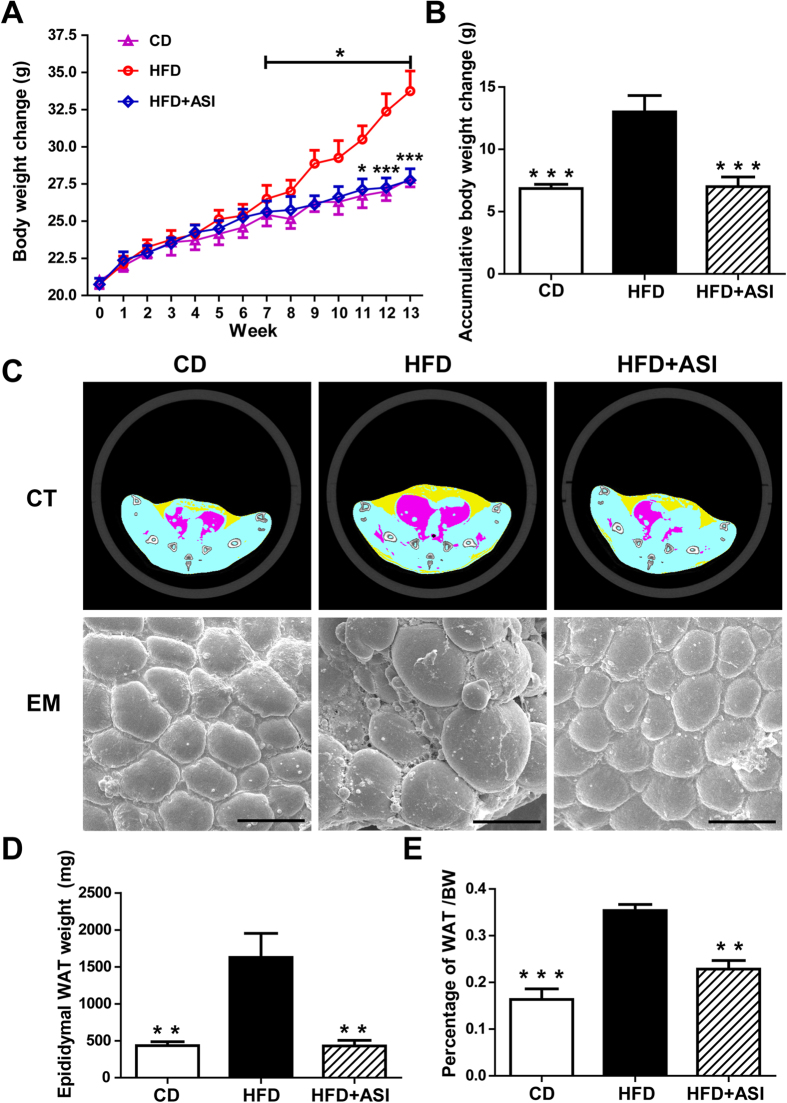
ASI prevented weight gain and reduced adipose tissue in HFD fed mice. (**A**) ASI administered at 25 mg/kg/d for 13 weeks prevented weight gain of HFD fed mice. Compared with HFD fed group mice, ASI treated HFD fed mice showed significantly lower increase in body weight from week 7 to week 13. N = 7–8/group. (**B**) ASI treated HFD fed mice displayed lower accumulative body weight change. N = 7–8/group. (**C**) ASI treated HFD fed mice accumulated less fat in subcutaneous (yellow) and visceral (purple) tissues. In addition, adipocytes in ASI treated mice kept the regular size as that in CD group mice as observed under scanning electron microscopy. Scale bar equals to 100 μm. (**D**) ASI treated mice had less epididymal WAT. N = 4–5/group. (**E**) ASI treatment reduced the ratio of WAT to body weight of HFD fed mice. N = 5/group. Data were presented as mean ± S.E.M. Compared with HFD group, *p < 0.05; **p < 0.01; ***p < 0.001.

C57BL/6 mice were randomly divided into three groups, i.e. CD group (fed with control diet), HFD group (fed with HFD) and ASI group (fed with HFD and treated with ASI). After 13 weeks of treatment, the average body weight of mice in HFD group was significantly higher than that in CD group (Fig. 1A). By contrast, the body weight of ASI group mice did not differ from that of the CD group (Fig. 1A). Similarly, the average accumulative body weight change of the mice in HFD group was remarkably higher than that in ASI group (Fig. 1B, *p* < 0.001). Body CT scan disclosed that subcutaneous and visceral fat accumulation in ASI group mice was markedly less than that in HFD group ones (Fig. 1C). The scanning electron microscope pictures of white adipose tissues (WAT) demonstrated that the adipocytes in mice of HFD group were prominently bigger than that in both ASI and CD groups (Fig. 1C). Similarly, HFD significantly elevated the epididymal adipose weight and increased the ratio of adipose tissue to body weight in mice compared with the CD (Fig. 1D,E, *p* < 0.01 and *p* < 0.001), the effect of which was significantly counteracted by ASI treatment (Fig. 1D,E).

### ASI improved lipid parameters in HFD fed mice

**Figure 2:**
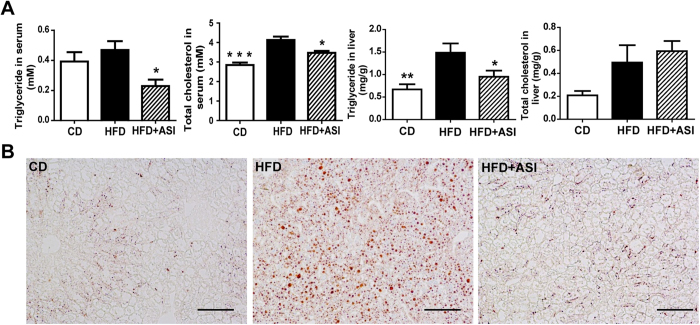
ASI treatment improved lipid parameters in HFD fed mice. (**A**) ASI administration reduced serum triglyceride, serum total cholesterol and liver triglyceride levels in HFD fed mice. N = 4/group. Data were presented as mean ± S.E.M. Compared with HFD group, *p < 0.05; **p < 0.01; ***p < 0.001. (**B**) ASI reduced lipid droplets in liver of HFD fed mice. Scale bar equals to 100 μm.

To determine the effect of ASI on dyslipidemia, the lipid levels in serum and liver were measured, respectively (Fig. 2A). Compared with the CD group, the HFD group mice displayed significantly increased levels of fasting serum TC (*p* < 0.001) and liver TG (*p* < 0.01), while serum TG and liver TC levels did not change. ASI administration significantly alleviated the elevation of serum TG (*p* < 0.05), TC (*p* < 0.05), and liver TG (*p* < 0.05) levels, but did not affect liver TC level. Accordingly, Oil Red O staining exposed that ASI treatment could effectively reduce lipid droplets in liver (Fig. 2B).

### ASI suppressed appetite and decreased respiratory quotient in HFD-fed mice

**Figure 3:**
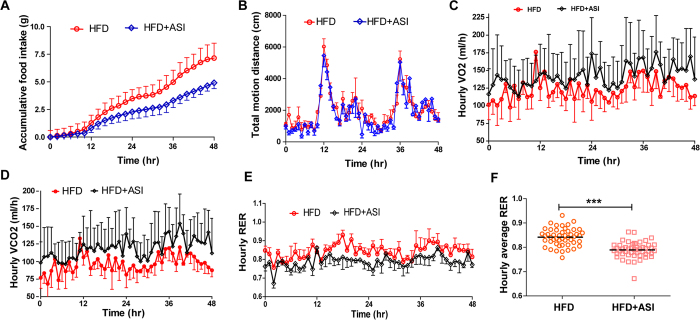
ASI suppressed appetite and decreased respiratory quotient in HFD-fed mice as measured in metabolic chamber. (**A**) ASI treated mice showed reduced food intake. N = 4/group. (**B**) ASI treatment did not change motor activity. N = 4/group. (**C**) ASI treatment elevated VO_2_ of HFD fed mice. (**D**) ASI treatment elevated VCO_2_ of HFD fed mice. (**E**) ASI treated mice displayed lower hourly RER during two days. N = 4/group. (**F**) ASI reduced hourly average RER of HFD fed mice. Data were presented as mean ± S.E.M. ***p < 0.001.

By the end of the weekly weight monitoring, mice were habituated in metabolic chambers for the measurement of food intake and other metabolic activities. In contrast to the HFD mice, ASI treated mice showed relatively suppressed food intake (Fig. 3A). However, there were no significant differences in the motion activity between ASI group and HFD group mice (Fig. 3B). As shown in Fig. 3C,D, ASI treated DIO mice displayed relatively elevated VCO_2_ and VO_2_, suggesting increased energy expenditure. And ASI decreased the amplitude of circadian respiratory exchange ratio (RER) in the HFD-fed mice (Fig. 3E, *p* < 0.05). Furthermore, there was significant difference in the hourly average RER between the two groups (Fig. 3F, *p* < 0.001), suggesting that ASI preferentially promoted oxidation of fatty acid rather than carbonhydrate in HFD-fed mice.

### ASI modulated expressions of genes associated with thermogenesis in HFD fed mice

**Figure 4:**
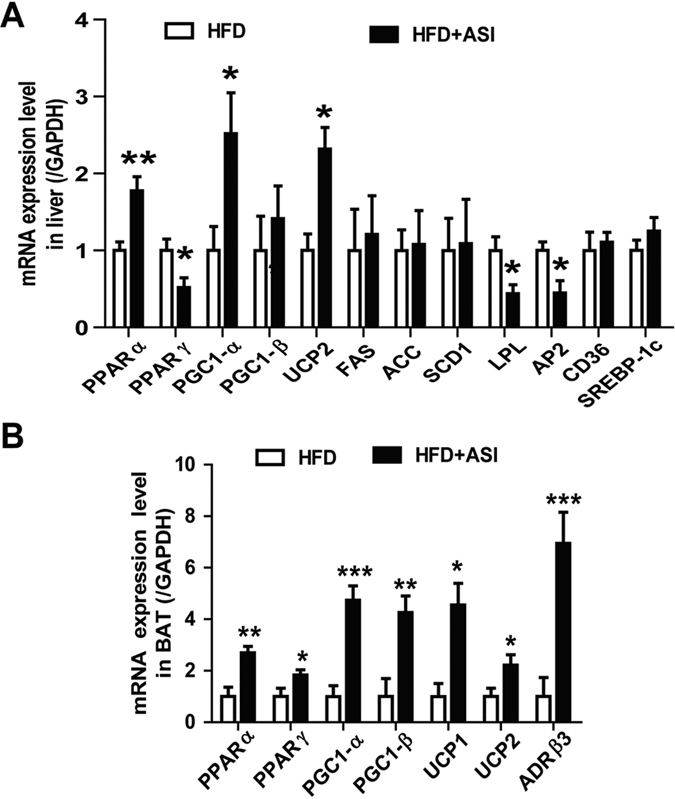
ASI modulated expression levels of genes associated with thermogenesis in liver and BAT of HFD fed mice. N = 6–8/group. Data were presented as mean ± S.E.M. Compared with HFD group, *p < 0.05; **p < 0.01; ***p < 0.001.

Liver and brown fat tissues (BAT) play important role in the maintenance of energy homeostasis. In liver, a series of genes involved in thermogenesis were examined. As shown in Fig. 4A, ASI treatment stimulated PPARα, PGC1-α, and UCP2 gene expression but down-regulated that of PPARγ, LPL and AP2. In BAT, thermogenesis related genes such as ADRβ3, PPARα, PPARγ, PGC-1α, PGC-1β, UCP1 and UCP2 were all increased significantly by ASI treatment. All of these results implicated that ASI actively participated in the modulation of energy homeostasis (Fig. 4B).

### ASI improved lipid parameters and modulated expressions of genes involved in thermogenesis in db/db mice

**Figure 5:**
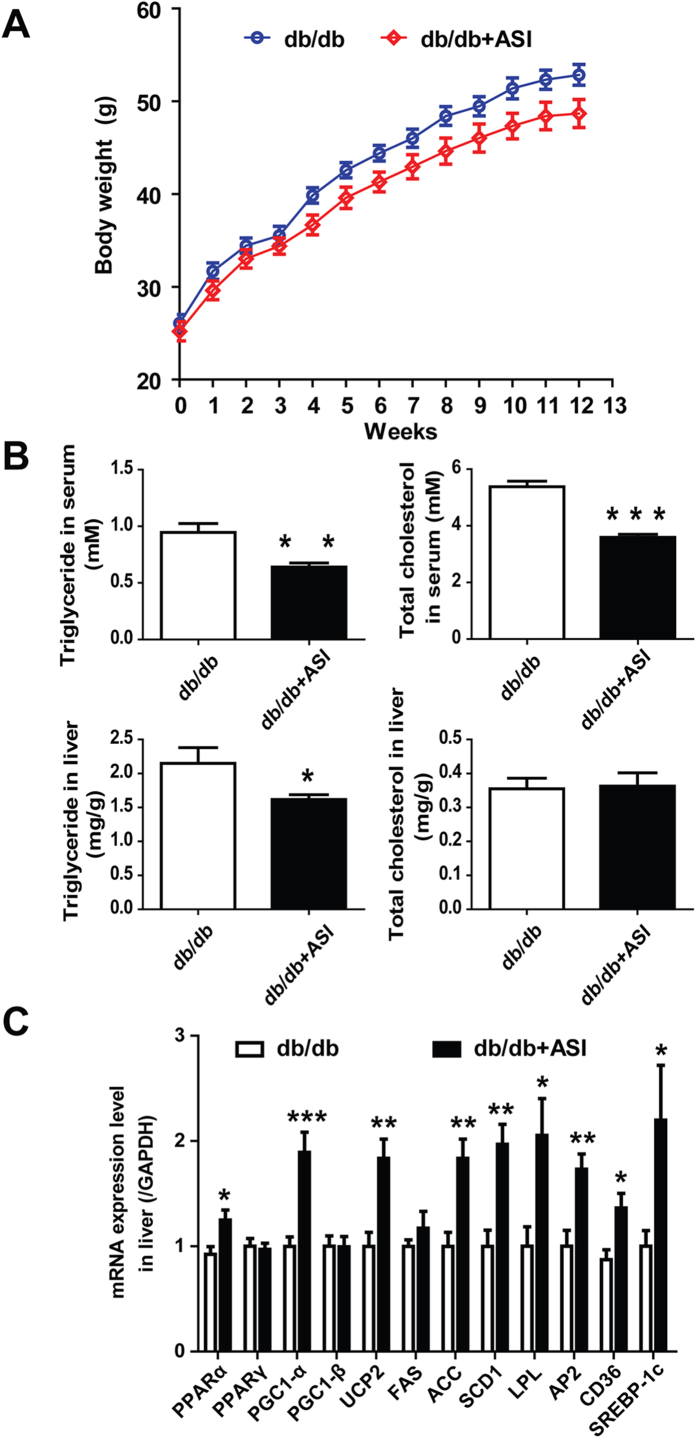
ASI attenuated hyperlipemia and up-regulated gene expressions associated with thermognesis in db/db mice. (**A**) ASI treatment did not reduce body weight gain of mice significantly. (**B**) ASI treatment decreased serum TG and TC, and liver TG in mice. (**C**) ASI modulated gene expressions associated with thermogenesis in liver. N = 6–8/group. Data were presented as mean ± S.E.M. Compared with db/db group, *p < 0.05; **p < 0.01; ***p < 0.001.

To examine if ASI could prevent obesity in other obese animal models, the leptin receptor deficient db/db mice were used. To our surprise, as shown in Fig. 5A, ASI treatment for 13 weeks did not suppress body weight gain of the mice significantly. However, as it did in HFD mice, ASI deceased serum TG and TC as well as liver TG in db/db mice (Fig. 5B, *p* < 0.01, *p* < 0.001 and *p* < 0.05). Furthermore, ASI increased gene expressions of PPARα, PGC1-α, UCP2, ACC, SCD1, LPL, AP2, CD36 and SREBP-1c in liver (Fig. 5C, *p* < 0.05, *p* < 0.01 or *p* < 0.001). The results indicated that ASI treatment modulated lipid metabolism and thermogenesis in db/db mice without affecting body weight gain.

### ASI enhanced expression of leptin and its receptors as well as appetite-associated genes in HFD fed mice

**Figure 6:**
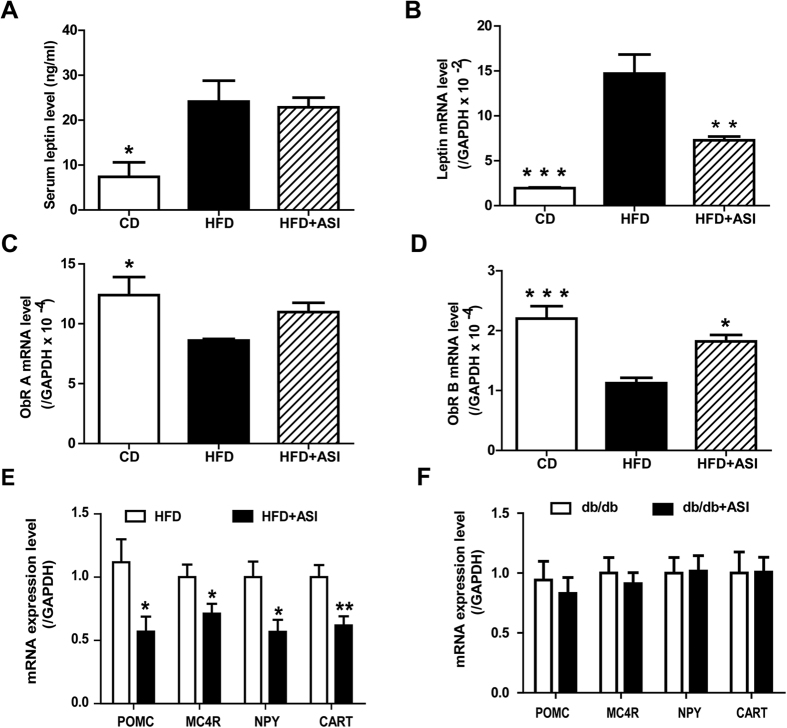
ASI enhanced leptin sensitivity in HFD fed mice. (**A**) Effect of ASI on serum leptin level. N = 4–6/group. (**B**) Effect of ASI on leptin mRNA expression level in WAT. N = 4–6/group. (**C,D**) Effect of ASI on hypothalamic ObR A and B mRNA expressions. N = 4–6/group. (**E**) Effect of ASI on hypothalamic genes associated with appetite in HFD fed mice. N = 4–6/group. (**F**) ASI treatment did not change mRNA expression levels of hypothalamic genes involved in regulation of appetite in db/db mice. N = 6/group. Data were presented as mean ± S.E.M. Compared with HFD group, *p < 0.05; **p < 0.01; ***p < 0.001.

Leptin is an adipocyte-derived hormone that suppresses appetite mainly through its action on a subset of hypothalamic neurons^23^,^24^. Considering ASI suppressing appetite, serum leptin, and mRNA expressions of leptin in WAT and leptin receptors in hypothalamus were analyzed, respectively. Compared with the CD group, the HFD group displayed significantly increased serum leptin and elevated leptin mRNA expression in WAT (Fig. 6A,B, *p* < 0.05 and *p* < 0.001), suggesting leptin resistance in HFD-fed mice. Compared with HFD mice without treatment, ASI treatment did not decrease serum leptin level but reduced leptin mRNA expression (*p* < 0.05) in HFD fed mice. In terms of leptin receptor, HFD caused significantly down-regulation of ObRa and ObRb mRNA expressions in hypothamus (Fig. 6C,D, *p* < 0.05 and *p* < 0.001). By contrast, ASI treatment kept relatively higher hypothalamic ObRa and ObRb mRNA expression levels (*p* < 0.05) in HFD fed mice.

Since leptin has been suggested to influence the expressions of other proteins regulating appetite such as pro-opio-melanocortin (POMC)-derived peptides, melanin concentrating hormone receptor 4 (MC4R), neuropeptide Y (NPY) and cocaine- and amphetamine-regulated transcript (CART) in hypothalamus, the effect of ASI on these genes were also investigated. As shown in Fig. 6E, ASI treatment significantly down-regulated mRNA expressions of all of these genes in HFD mice. These results demonstrated that ASI increased leptin sensitivity in HFD fed mice.

However, the mRNA expression levels of POMC, MC4R, NPY and CART in hypothalamus were not altered by ASI treatment in db/db mice (Fig. 6F). The results suggested that ASI prevented body weight gain and suppressed appetite associated gene expression through ObR.

### ASI enhanced leptin signaling in neuronal cell line SH-SY5Y

**Figure 7:**
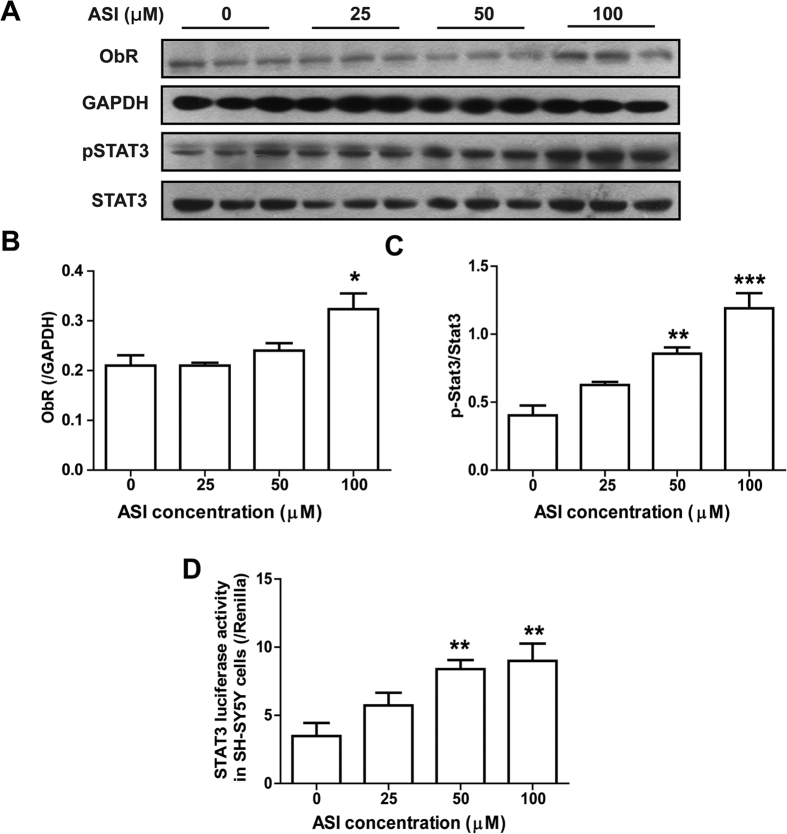
ASI enhanced leptin signaling in SH-SY5Y cells. (**A–C**) ASI dose-dependently regulated ObR expression and STAT3 activation. (**D**) ASI dose-dependently elevated luciferase activity in cells transfected with STAT3 luciferase reporter plasmid. N = 4/group. Data were presented as mean ± S.E.M. Compared with control, *p < 0.05; **p < 0.01; ***p < 0.001.

To determine if ASI could directly activate leptin signaling in neuronal cells, the expression of ObR and activation of STAT3 in SY-SY5Y cells were investigated. As shown in Fig. 7A,B, ASI induced dose-dependently ObR expression after treated for 24 h. Moreover, ASI stimulation enhanced the activation of STAT3 as more phosphorylated STAT3 was found particularly in cells treated with higher concentration of ASI (Fig. 7A,C). To confirm the activation of STAT3 by ASI, STAT3-luciferase assay was conducted. As displayed in Fig. 7D, ASI induced significantly elevated STAT3-luciferase activity in SH-SY5Y cells in a dose-dependent manner. These results revealed that ASI probably alleviated leptin resistance by comprehensive modulation of ObR/STAT3 signaling transduction in neuronal cells.

## Discussion

Obesity is a chronic disease resulting from an imbalance between energy intake and expenditure, in which leptin resistance in most cases is responsible for the disturbance of body weight control^11^. Leptin resistance or impaired leptin synthesis, signaling cascades or sensitivity can disrupt peripheral physiological functions of leptin including lipid and carbohydrate metabolism. In the present study, we showed that ASI, a natural saponin molecule, could effectively reduce body weight gain, lessen adipocyte size and decrease adipose tissue weight of HFD fed mice with notably diminished lipid contents. Further study demonstrated that ASI suppressed appetite and decreased the respiratory quotient in the HFD-fed mice probably by increasing leptin sensitivity.

Excessive body fat accumulation is the prominent characteristic of obesity. Activation of leptin receptor increases sympathetic nervous system activity, resulting in enhanced energy expenditure in adipose tissue^25^. Due to leptin resistance, although most obese humans and rodents have very high level of circulating leptin, hyperleptinemia can not reduce appetite or accelerate energy expenditure. Consistently, in the present study, HFD feeding for 13 weeks induced high leptin expressions at both gene and protein levels but decreased leptin receptor expression in mice, indicating the development of leptin resistance. As a result, increased TG, TC, WAT, and enlarged adiocytes, as well as hyperphagia were found in HFD-induced obese mice. However, ASI administration could efficiently reverse HFD induced obese parameters in mice. Meanwhile, ASI accelerated ObR synthesis at mRNA level under leptin repletion in hypothalamus, the major site for leptin sensing^26^. In agreement with *in vivo* data, ASI could also increase ObR synthesis in neuronal cell line SH-SY5Y *in vitro* in addition to the enhancement of STAT3 activation. However, to our surprise, ASI showed no obvious inhibitory effect on serum leptin level of HFD mice. Generally, it is thought that leptin levels track with adiposity. However, it is also suggested that a large portion of the interindividual variability in plasma leptin concentration is independent of body fatness^27^, since it is also produced in brown fat tissue, placenta, ovary, skeletal muscle, stomach, pituitary gland, bone marrow and liver other than adipose tissue^28^. As displayed by our result, ASI decreased leptin mRNA expression in white adipose tissue. However, we did not examine whether ASI elevated leptin mRNA expressions in other tissues. Therefore, it is still possible that ASI enhanced leptin synthesis in other tissues that finally resulted in the high serum leptin level in the relatively lean mice. Thus, all of these results implicated improved leptin sensitivity by ASI treatment in HFD fed mice.

In hypothalamus two populations of leptin-responsive neurons exert opposite functions on food intake are found. One population expresses α-melanocyte-stimulating hormone (α-MSH) originated from POMC precursor with anorexigenic effect. The other population produces NPY and AgRP with orexigenic effect, which was suppressed by leptin stimulation. ASI has been indicated to be able to pass through blood-brain barrier in rodents when administrated intravenously^29^. Consistently, our unpublished data also revealed that ASI could be detected in brain parenchyma of experimental autoimmune encephalomyelitis mice. In present experiments, ASI treatment inhibited mRNA expressions of POMC, MC4R (the receptor for α-MSH), NPY and CART in hypothalamus of DIO mice. In terms of protein expression level, ASI enhanced MC4R but reduced NPY in hypothalamus of DIO mice (Fig. S1). In leptin receptor deficient db/db mice, the regulatory effect of ASI on POMC, MC4R, NPY and CART was abolished. Meanwhile, ASI treated db/db mice showed no difference in food intake with the untreated ones (unpublished data). The results suggested that ASI modulated these hypothalamic genes dependent on leptin signaling pathway.

Liver plays an important role in the maintenance of energy homeostasis, which is regulated by leptin in terms of hepatic gluconeogenesis and insulin sensitivity. Leptin resistance or impaired leptin function results in hyperglycemia, hyperinsulinemia and hyperlipidemia in liver^30^. Opposite to WAT, the main site for metabolic energy storage, BAT is a specialized thermogenic organ that produces heat during cold or high calorie diet exposure^31^. In agreement with previous reports, in our study, HFD fed mice showed significant hyperlipidemia. ASI administration deceased lipid droplets in liver of HFD-fed mice accompanied with significant modulation of gene expressions of PPARα, PPARγ, PGC1-α, UCP2, LPL and AP2. In BAT, similarly, ASI also up-regulated gene expressions of ADRβ3, PPARα, PPARγ, PGC1-α, PGC1-β, UCP1 and UCP2. While in db/db mice, ASI increased gene expressions of PPARα, PGC1-α, UCP2, ACC, SCD1, LPL, AP2, CD36 and SREBP-1c in liver. All of the aforementioned genes in liver or BAT are actively involved in the maintenance of energy homeostasis by modulation of lipogenesis and energy expenditure^32^,^33^,^34^,^35^,^36^,^37^, suggesting complicated network of thermogenesis regulated by ASI. However, ASI showed different regulatory pattern in hepatic mRNA expression of LPL and AP2 in HFD-fed mice from that in db/db mice. The shifted regulatory pattern reflected that ASI exerted its hypolipidemic function associated with leptin signaling pathway.

Loss of adipose tissue is associated with an increase in fat oxidation. Leptin has been suggested to trigger fuel selection from carbohydrate oxidation to fat oxidation^38^. In metabolic chamber, respiratory quotient is a parameter as an indicator for the fuel selection. Our data displayed that ASI treatment resulted in the decrease of RER in HFD fed mice without alteration of circadian motion activity, implicating that ASI prompted fat oxidation, therefore, decreased its accumulation in HFD-fed mice probably through the action of leptin.

In summary, our results demonstrated that ASI prevented the process of obesity, lowered lipid contents, and enhanced fat oxidation in obese mice by interfering thermogenic network and ameliorating leptin resistance.

## Materials and Methods

### High-fat diet challenge and drug treatment

Six-weeks old male C57BL/6 mice were group-housed and fed with either 45% HFD (D12451, Research Diets, New Brunswick, NJ) or control diet (CD, D12450B). Six-weeks old male db/db mice were provided by Model Animal Research Center of Nanjing University (Nanjing, China), group-housed and fed with normal chow diets. Intraperitoneal administration of ASI (25 mg/kg) was given daily to the mice from the beginning of the experiments and continued for 13 weeks. The dose of ASI was determined based on our preliminary experiment. Body weight (BW) was monitored weekly and the fat composition was examined by a CT scanner (Latheta LCT200, EchoMRI, USA ) at 2-mm intervals from the diaphragm to the bottom of the abdominal cavity. Food intake, respiratory quotient (RQ) and motor activity were measured by Oxymax metabolic chambers (MotoRater System; TSE Systems, Bad Homburg, Germany). The mice were placed into the single-caged metabolic chamber to acclimatize one day before the initiation of data collection for two consecutive days.

All animal experiments were performed according to the protocols approved by Animal Care and Use Committee of Shanghai University of Traditional Chinese Medicine, which complies with international rules and policies. Ethics in accordance with the ARRIVE (Animal Research: Reporting *In Vivo* Experiments) guidelines were followed in the animal experiments and were approved by the Animal Care and Use Committee of Shanghai University of Traditional Chinese Medicine. All efforts were made to minimize suffering and reduce the number of animals used.

### Triglyceride, cholesterol measurement and ELISA

Mice were fasted for 12 h prior to collect blood and liver samples for further biochemical analysis. Levels of triglyceride (TG) and total cholesterol (TC) were determined using respective kits from Jiancheng Bioengineering Institute (Nanjing, China). The serum concentration of leptin was measured with ELISA kit from Merck Millipore Company (Massachusetts, USA).

### Cell culture and treatment

Neuronal cell line SH-SY5Y cells were maintained in DMEM/F-12 medium supplemented with 10% fetal bovine serum (FBS) at 37 °C in a humidified incubator supplied with 5% CO2. After seeded in 6-well plate at 5 × 10^5^ cells/ml and cultured without FBS overnight, the cells were treated with ASI (0, 25, 50 and 100 μM) for 24 h. Thereafter, they were harvested with CelLytic^TM^ MT mammalian tissue lysis reagent (Sigma, USA) supplemented with protease and phosphatase inhibitors (Sigma) followed by sonication, and stored at −80 °C for further western blotting analysis.

### Quantitative PCR

Total RNA from mouse hypothalamus, liver and fat tissues was extracted using Trizol in accordance with the manufacturer’s manual (Life Technologies, Grand Island, NY, USA). The first strand cDNA was reverse transcribed with kit from Life Technologies. The expressions of respective genes were quantified by real-time PCR using SYBR Green Master Mix kit (Life Technologies), which were normalized to that of glyceraldehyde-3-phosphate dehydrogenase (GAPDH) in the same sample. The sequences of primers used were listed in Table 1.

### Histological examinations

The architecture of abdominal fat tissue was examined by scanning electron microscopy referred to the previous method of Chun *et al.*^39^. For Oil Red O staining, liver sections were fixed in 10% formaldehyde, incubated in 0.2% Oil Red O in isopropanol for 10 min, rinsed with 60% isopropanol for 10 min followed by elution with tap water for another 10 min.

### Western blotting analysis

Proteins from cells or tissues were extracted by sonication in CelLytic^TM^ MT mammalian tissue lysis reagent with protease and phosphatase inhibitor cocktails. After centrifugation at 12,000 rpm for 10 min at 4 °C, the supernatant of the lysate was collected and the protein concentration was determined by BCA method. Totally, thirty microgram of proteins from each sample were separated by 10% SDS-PAGE and transferred onto PVDF membranes. After blocked with 5% skim milk, the membranes were incubated with respective primary antibodies against phospho-Stat3 (p-Tyr705, CST, 9145S), Stat3 (CST, 9139), ObR (Abcam, ab177469), GAPDH (CST, 5174), and, sequentially, secondary antibodies conjugated with horseradish peroxidase (Life Technologies). The signal was visualized with the ECL prime kit (GE Healthcare, UK). Relative quantification of the bands was performed by using Image J 1.46r (NIH, USA).

### STAT3-luciferase assay

To determine the effect of ASI on leptin signaling, the STAT3-luciferase based method was employed as described previously^40^,^41^. The pAH-luc-STAT3 reporter plasmid was bestowed by Dr. Weihong Pan at Pennington Biomedical Research Center (Baton Rouge, LA, USA). The Renilla-luciferase reporter plasmid was provided by Dr. Wei Dou at Institute of Chinese Materia Medica (SHUTCM, Shanghai, China). SH-SY5Y cells were seeded in 24-well plates at 5 × 10^5^ cells/ml and transfected with respective plasmids using lipofectamine 3000 (Life Technologies) in serum free medium according to the manual’s instructions. Six hours later, the cells were serum-starved and treated with ASI (0, 25, 50, 100 μM) for 24 h. Afterwards, the cells were harvested and subjected to STAT3-luciferase assay with Dual-Luciferase Reporter Gene Assay kit (Promega, WI, USA).

### Statistical analysis

All data were presented as mean ± S.E.M. Differences in body weight, food intake and respiratory quotient between HFD and CD mice were analyzed with repeated-measures ANOVA, followed by Tukey’s post hoc test. All the other comparisons among groups were determined by one-way ANOVA with Dunnett’s post hoc test. Difference between two groups was analyzed with un-paired t-test. Differences were regarded as statistically significant as P value < 0.05.

## Additional Information

**How to cite this article**: Wu, H. *et al.* Astragaloside IV improves lipid metabolism in obese mice by alleviation of leptin resistance and regulation of thermogenic network. *Sci. Rep.* **6**, 30190; doi: 10.1038/srep30190 (2016).

## Supplementary Material

Supplementary Information

## Figures and Tables

**Table 1:** Sequences of primers for quantitative PCR.

Gene name	Forward Primer	Reverse Primer
ObRa	GAAGTCTCTCATGACCACTACAGATGA	TTGTTTCCCTCCATCAAAATGTAA
ObRb	GCATGCAGAATCAGTGATATTTGG	CAAGCTGTATCGACACTGATTTCTTC
Leptin	ATGTGCTGCAGATAGCCAATGA	AGGGAGCAGCTCTTGGAGAAG
POMC	ATGCCGAGATTCTGCTACAGT	TCCAGCGAGAGGTCGAGTTT
MC4R	CCCGGACGGAGGATGCTAT	TCGCCACGATCACTAGAATGT
NPY	ATGCTAGGTAACAAGCGAATGG	TGTCGCAGAGCGGAGTAGTAT
CART	CCCGAGCCCTGGACATCTA	GCTTCGATCTGCAACATAGCG
PPARα	AGAGCCCCATCTGTCCTCTC	ACTGGTAGTCTGCAAAACCAAA
PPARγ	TCATGACCAGGGAGTTCCTC	CAGCAGGTTGTCTTGGATGT
PGC-1α	TATGGAGTGACATAGAGTGTGCT	CCACTTCAATCCACCCAGAAAG
PGC-1β	CCTTCCACCTGAGCTATGGA	TATTGGAAGGGCCTTGTCTG
UCP1	GTACCAAGCTGTGCGATGTC	TGGTCTCCCAGCATAGAAGC
UCP2	ACTGTGCCCTTACCATGCTC	CTGGGCAGAGGATGAAGAAA
ADRβ3	CCTTCCGTCGTCTTCTGTGT	AGAAGATGGGGATCAAGCAA
FAS	GGAGGTGGTGATAGCCGGTAT	TGGGTAATCCATAGAGCCCAG
ACC	ATGGGCGGAATGGTCTCTTTC	TGGGGACCTTGTCTTCATCAT
SCD1	TTCTTGCGATACACTCTGGTGC	CGGGATTGAATGTTCTTGTCGT
LPL	GGGAGTTTGGCTCCAGAGTTT	TGTGTCTTCAGGGGTCCTTAG
AP2	CCCGCATGGAGGGTGTATG	TGGAGGGATCACGAGCTTGAA
CD36	TTTGGAGTGGTAGTAAAAAGGGC	TGACATCAGGGACTCAGAGTAG
SREBP-1c	GATGTGCGAACTGGACACAG	CATAGGGGGCGTCAAACAG
GAPDH	ATGTGTCCGTCGTGGATCTGA	ATGCCTGCTTCACCACCTTCT
