# Supplementary material for: Astragaloside IV improves lipid metabolism in obese mice by alleviation of leptin resistance and regulation of thermogenic network
Source: Sci Rep. 2016 Jul 22;6:30190. doi: 10.1038/srep30190 (PMC4957129; doi:10.1038/srep30190)

Title page

Title

Astragaloside IV improves lipid metabolism in obese mice by alleviation of leptin resistance and regulation of thermogenic network

Hui Wu[1,#], Yan Gao[1,#], Hai-Lian Shi[1], Li-Yue Qin[1], Fei Huang[1], Yun-Yi Lan[1], Bei-Bei Zhang[1], Xiao-Jun Wu[1,*], Zhi-Bi Hu[1,*]

Running title: ASI enhances leptin sensitivity & theromogenesis

[1]Shanghai Key Laboratory of Complex Prescription, The Ministry of Education (MOE) Key Laboratory for Standardization of Chinese Medicines, Institute of Chinese Materia Medica, Shanghai University of Traditional Chinese Medicine, Shanghai, 201203, China

Corresponding authors[*]

Xiaojun Wu and Zhibi Hu, Institute of Chinese Materia Medica, Shanghai University of Traditional Chinese Medicine, 1200 Cailun Road, Shanghai, 201203, China. E-mail: xiaojunwu320@126.com(XJ Wu), huzhibi@hotmail.com(ZB Hu); Tel.: +86 21 51322578; Fax: +86 21 51322505.

[#]These authors contributed equally to this work.

Supplementary figure legends

Figure S1. ASI enhanced MC4R but reduced NPY expression in hypothalamus of DIO mice as exposed by western blotting analysis.

Fig.S1

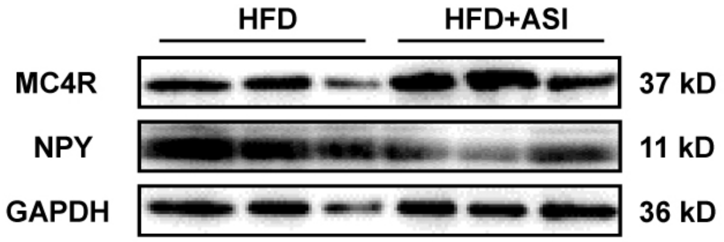

The original bands for all the Figures in the main manuscript and Fig.S1

Figure 7A ObR

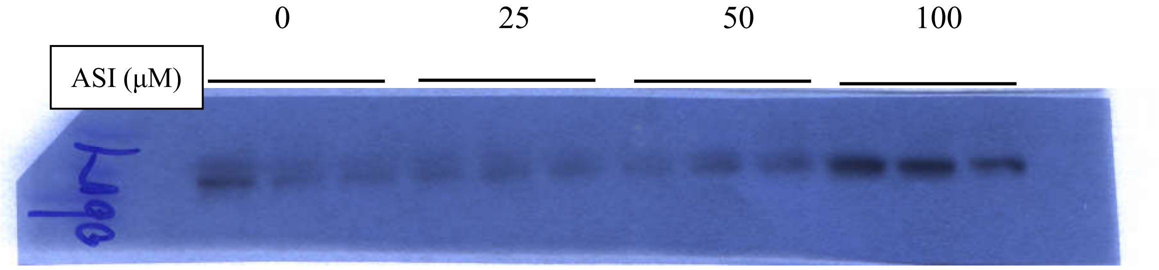

Figure 7A GAPDH

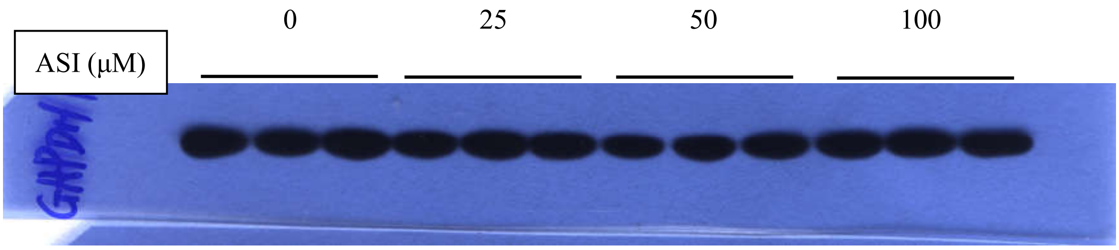

Figure 7A pSTAT3

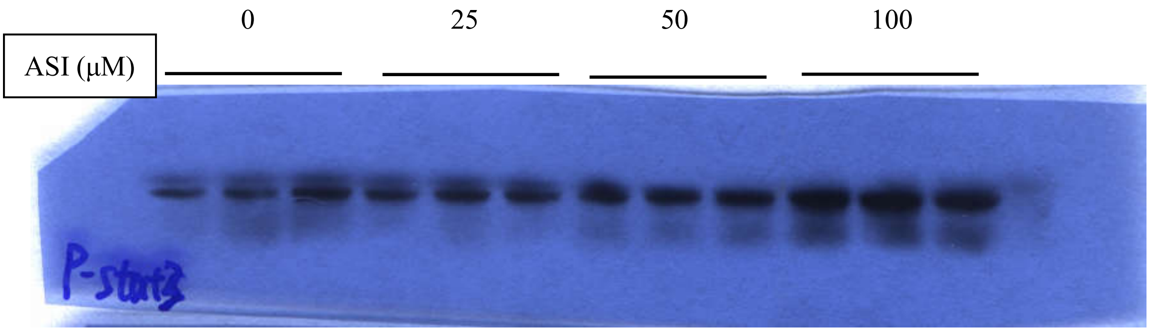

Figure 7A STAT3

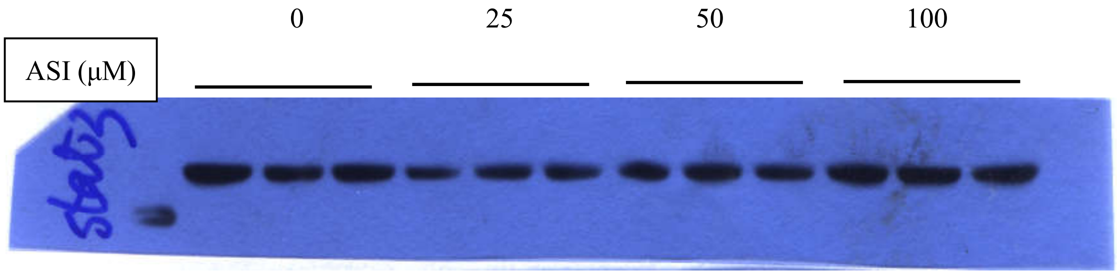

Figure S1 MC4R

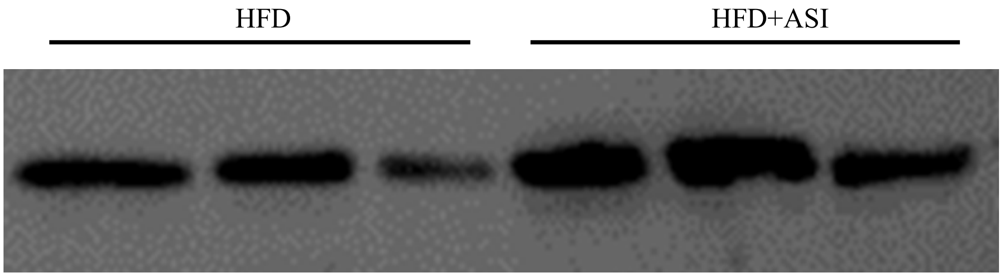

Figure S1 NPY

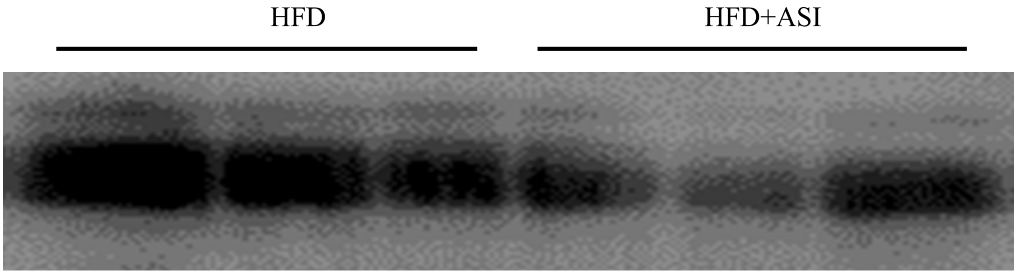

Figure S1 GAPDH

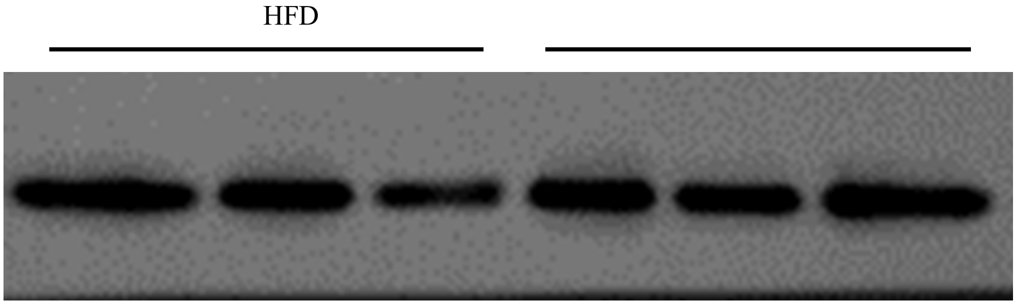

Supplement: Supplementary Information [file srep30190-s1.pdf]
